# Does cartilage volume measurement or radiographic osteoarthritis at baseline independently predict ten-year cartilage volume loss?

**DOI:** 10.1186/s12891-016-0900-7

**Published:** 2016-02-02

**Authors:** Andrew McBride, Hussain Ijaz Khan, Dawn Aitken, Louisa Chou, Changhai Ding, Leigh Blizzard, Jean-Pierre Pelletier, Johanne Martel-Pelletier, Flavia Cicuttini, Graeme Jones

**Affiliations:** Menzies Institute for Medical Research, University of Tasmania, Medical Science 1 Building, Private Bag 23 17-Liverpool Street, Hobart, 7000 Australia; Department of Epidemiology and Preventive Medicine, Monash University, Melbourne, Australia; Osteoarthritis Research Unit, University of Montreal Hospital Research Centre (CRCHUM), Montreal, QC Canada

**Keywords:** Knee, Osteoarthritis, Cartilage volume, Magnetic resonance imaging, Radiographs

## Abstract

**Background:**

The aim of this study was to examine whether cartilage volume as measured by MRI and radiographic osteoarthritis (OA) at baseline predict cartilage volume loss over ten years independent of each other and other structural co-pathologies.

**Methods:**

219 participants [mean-age 45(26–61); 57 % female] were studied at baseline and ten years. Approximately half were the adult offspring of subjects who underwent knee replacement for OA and the remainder were randomly selected controls. Joint space narrowing (JSN) and osteophytes were assessed on radiographs and cartilage volume (tibiofemoral), cartilage defects, bone marrow lesions and meniscal tears/extrusion were assessed on MRI.

**Results:**

Mean absolute and percentage per annum cartilage volume loss was 1284 mm^3^ and 1.91 % respectively in the medial compartment and 1007 mm^3^ and 1.38 % respectively in the lateral compartment. Higher baseline tibiofemoral cartilage volume was independently associated with greater absolute cartilage volume loss in both medial (β(95 % CI) = −300 (−399,−200)) and lateral (β = −338 (−443,−233)) compartments and percentage per annum loss in the lateral compartment(β = −0.15 (−0.29, −0.01)). Baseline JSN and osteophytes were associated with cartilage volume loss in the univariable analysis, however these associations did not persist after adjustment for other structural co-pathologies.

**Conclusion:**

Cross-sectional cartilage volume measurement independently predicts cartilage volume loss over 10 years and can be used to identify fast progressors in clinical trials. Radiographic JSN and osteophytes on the other hand are a reflection of other co-pathologies assessed on MRI and do not independently predict cartilage volume loss over 10 years.

## Background

Osteoarthritis (OA) is characterised by whole joint abnormalities including gradual cartilage volume loss [1]. Recent studies suggest that a cascade of structural changes occur in OA that involve sub-chondral bone expansion, bone marrow lesions (BMLs), meniscal tears, extrusion and eventually gradual loss of articular cartilage [1–7]. Loss of cartilage volume starts around the age of 40 years when radiographic changes are uncommon. [8] In early OA cartilage swelling appears to precede volume loss [6, 9]. This is supported by longitudinal evidence that higher baseline cartilage volume is associated with greater volume loss over a two-year period in early OA [2]. In patients with established OA, lower baseline cartilage volume appears to predict loss over a similar period [3].

Radiographic osteoarthritis (ROA) score has also been found to predict cartilage volume loss [10]. Whether this association is due to the presence of osteophytes or joint space narrowing (JSN) remains controversial. One study found that both JSN and osteophytes act as independent predictors of volume loss in a cohort of randomly selected older adults from community over a two-year period [10]. Other studies have shown that knees with definite osteophytes but without JSN do not show significantly greater rates of cartilage volume loss compared to healthy knees over a one-year period [11]. Similarly studies have shown that both presence [12, 13] and severity of ROA [11] is associated with cartilage thickness loss as well. To our knowledge no papers have looked at the association between ROA scores in early disease and volume loss over a ten-year period.

The aim of this study, therefore, was to examine whether cartilage volume as measured by MRI and ROA at baseline predict cartilage volume loss over ten years independent of each other and other structural co-pathologies.

## Methods

### Study subjects

This study was conducted as part of the Offspring study, which is an ongoing population-based study. The Offspring study began in southern Tasmania (primarily in the city of Hobart) in June 2000. Matched sampling was used to recruit the study participants (mean age 45 (26–61) years; 58 % females). Half of the participants were the adult offspring of patients who had a knee replacement performed for idiopathic knee OA at any Hobart hospital from 1996 to 2000 [5]. The diagnosis was confirmed by reference to the medical records of the orthopaedic surgeon and the original radiographs when possible. The other half were age and sex matched controls, randomly selected from the population with no history of knee OA in either parent. Controls were randomly selected from the electoral roll in southern Tasmania (population 229,000), a comprehensive population listing. This study includes data from the baseline visit, 2 year and 10 year follow up.

The Southern Tasmanian Health and Medical Human Research Ethics Committee approved the protocol, and written informed consent was obtained from all participants. Participants were excluded if they had a contraindication to MRI (including metal sutures, presence of shrapnel, iron filing in eye, or claustrophobia). Participants were also excluded if they had undergone a knee replacement surgery or did so after the commencement of the study. Knee pain and knee injury were not a basis for exclusion.

### Anthropometrics

Weight was measured to the nearest 0.1 kg (with the subject’s shoes, socks, and bulky clothing removed), with a single pair of electronic scales (Delta Model 707; Seca, Munich, Germany) that were calibrated using a known weight at the beginning of each clinic session. Height was measured to the nearest 0.1 cm (with shoes and socks removed) using a stadiometer. Body mass index (BMI) was calculated as weight (kg)/height (m^2^).

## Magnetic resonance imaging

MRI of the right knee was performed as described previously [14–16]. All knees were imaged in the sagittal plane on a 1.5-T whole-body magnetic resonance unit (Picker International, USA) using a commercial transmit-receive extremity coil. The following image sequence was used: (i) a T1-weighted fat-suppressed 3D gradient-recalled acquisition in the steady state, flip angle 55°, repetition time 58 msec, echo time 12 msec, field of view 16 cm, 60 partitions, 512 × 512–pixel matrix, slice thickness of 1.5 mm without an interslice-gap; and (ii) a T2-weighted fat saturation 2D fast spin echo, flip angle 90°, repetition time 3067 ms, echo time 112 ms, field of view 16 cm, 15 partitions, 256 × 256 matrix, slice thickness of 4 mm with an interslice gap of 0.5–1.0 mm.

### Cartilage volume assessment

Knee cartilage volume was evaluated at baseline and 10 years by a trained observer on T1-weighted gradient echo MR images. Knee cartilage volume was determined by means of image processing on an independent workstation at baseline and follow up. The volumes of individual cartilage plates (medial tibia and femora, and lateral tibia and femora) were isolated from the total volume by manually drawing dis-articulation contours around the cartilage boundaries on a section by section basis. These data were then resampled by means of bilinear and cubic interpolation (area of 312 × 312 μm by 1.5 mm thickness, continuous sections) for the final three-dimensional rendering to calculate the cartilage volume.

Tibial cartilage volume was assessed using Osiris (University of Geneva, Switzerland) software as previously described [14, 17]. The coefficient of variation(CV) ranged from 2.1 to 2.2 % for intra-observer repeatability [18]. Femoral cartilage volume was determined using Cartiscope (ArthroLab, Montreal, Canada), as previously described [19, 20]. The CV was approximately 2 % for intra-observer and inter-scan repeatability [20]. Total cartilage volume was calculated as: tibial + femoral cartilage volume.

Absolute cartilage volume loss was calculated as: follow-up total cartilage volume - baseline total cartilage volume. Percentage per annum cartilage volume loss was calculated as: ((absolute cartilage volume loss/baseline cartilage volume)/time period between MRI acquisition at baseline and visit-3) × 100.

### Cartilage defects

Cartilage defects were assessed at baseline and 10 years on T1-weighted gradient echo MR images at the medial tibial, medial femoral, lateral tibial, and lateral femoral sites on a 0–4 scale, as previously described [16]: grade 0 = normal cartilage; grade 1 = focal blistering and intracartilaginous low-signal intensity area with an intact surface and base; grade 2 = irregularities on the surface or base and loss of thickness <50 %; grade 3 = deep ulceration with loss of thickness >50 %; and grade 4 = full-thickness chondral wear with exposure of subchondral bone. Intraobserver reliability (expressed as intraclass correlation coefficient (ICC)) ranged from 0.89 to 0.90. Interobserver reliability was assessed in 50 MR images and yielded an ICC of 0.85–0.90 [16].

### Meniscal tears

Meniscal tears were assessed by a trained observer on T1-weighted gradient echo and T2-weighted (side by side) MR images at visit-2 and 3 of the study as previously described [19]. The proportion of the menisci affected by a tear was scored separately (0–2 scale; 0 = absence of a tear, 1 = simple tear of different types: longitudinal, oblique, radial or horizontal, 2 = complex tear signifying loss > 50 % area of meniscal tissue) at the anterior, middle, and posterior horns (medially/laterally). Anterior, middle and posterior scores were summed to get medial and lateral meniscal tear scores. The intra- and inter-observer correlation coefficient ranged from 0.86 to 0.96 [20]. Meniscal tears were scored at visit-2 of the Offspring study, 2 years after the baseline visit.

### Meniscal extrusion

Meniscal extrusion was assessed by a trained observer on T1-weighted gradient echo MR images at baseline and 10 years as previously described [19]. The proportion of the menisci affected by a partial or full extrusion was scored separately (yes/no) at the anterior, middle, and posterior horns (medially/laterally). Anterior, middle and posterior scores were summed to get medial and lateral meniscal tear/extrusion scores. The intra- and inter-observer correlation coefficient ranged from 0.85 to 0.92 for meniscal extrusion [20].

### Bone marrow lesions

Bone marrow lesions (BMLs) were assessed on fat suppressed T2-weighted MR images as described previously [21]. BMLs were defined as areas of increased signal intensity in the sub-chondral bone at the medial tibial, medial femoral, lateral tibial, lateral femoral, superior patellar and inferior patellar sites. One trained observer scored the BMLs by measuring the maximum area of the lesion in a specific compartment. The observer manually selected the MRI slice with the greatest BML size. The BML with the highest score was used if more than one lesion was present at the same site. The ICC was 0.97. BMLs were scored at visit-2 of the Offspring study, 2 years after the baseline visit.

## Radiography

A standing anteroposterior semiflexed x-ray of the right knee was taken in all subjects at baseline and 10 years. The angle was kept to 10–15° by a purpose built goniometer. The tube to film and tube to tibial plateau angle was 90°. Daily quality assurance was performed on the equipment. Radiographs were scored individually for osteophytes and joint space narrowing (JSN), as described previously [22]. Each of the following four features was scored on a scale from 0 to 3 (0 = normal and 3 = severe): medial JSN, lateral JSN, medial osteophytes (femoral and tibial combined) and lateral osteophytes (femoral and tibial combined). Each score was arrived at by consensus with two readers (LC, AM) simultaneously assessing the radiograph with immediate reference to the Osteoarthritis Research Society International (OARSI) atlas [23]. ROA score was calculated by adding JSN (medial and lateral sites) and osteophytes (medial and lateral tibial and femoral sites) scores. A non-zero score in either JSN or osteophytosis was regarded as evidence of any ROA. Total ROA score had a possible range of 0–18. Reproducibility was assessed in 50 radiographs, two weeks apart, and yielded an agreement (linear weighted kappa value) of 0.87–1.00 for osteophytes and 0.94–1.00 for JSN (*p*-value < 0.001).

Readers for all the scans were either musculoskeletal radiologists with several years of experience in OA research or health professionals trained by musculoskeletal radiologists. Readers were not blinded to the chronological sequence of the radiographs and MRI scans.

## Statistical analysis

T-tests and chi-square tests were used to compare differences in means and proportions as appropriate when examining demographic, cartilage volume and radiographic data. Baseline characteristics of the participants were split into two groups for comparison using mean total cartilage volume loss (absolute) over 10 years: (i) less than mean total cartilage volume loss; (ii) greater than or equal to mean total cartilage volume loss.

Linear regression analysis was used to examine the association between baseline radiographic structures/cartilage and cartilage volume loss (absolute and percentage) over 10 years. β-coefficients were standardised to describe the association between baseline radiographic structures/cartilage volume and cartilage volume loss, so that cartilage volume loss was expressed as loss per standard deviation change in the predictor variables [21, 24, 25]. Multivariable analysis was adjusted for age, sex, BMI, offspring-control status, radiographic structures/cartilage volume at baseline and MRI structures which had a higher prevalence (or showed a similar trend) in participants with greater than or equal to mean total cartilage volume loss. All the associations between baseline JSN and cartilage loss were adjusted for baseline osteophytes and vice versa. Interactions terms were calculated to examine significant differences between the offspring and control groups.

Further sub-analyses looking at the association between baseline cartilage volume and/or ROA and absolute cartilage volume loss stratified by mean age was performed to look at the effect of advancing age on the associations described in the study.

To counter the effect of regression to the mean/tracking, when describing the association between baseline cartilage volume and cartilage volume loss, further sub-group analysis was done with baseline cartilage volume stratified by the mean value.

A *P*-value of less than 0.05 (two-tailed) was considered statistically significant. All statistical analyses were performed on Intercooled Stata 12.0 for windows (StataCorp LP).

## Results

Of the 371 participants included in the Offspring study, 219 between the ages of 26 and 61 years were followed up after 10 years. The characteristics of participants who were followed up compared to participants who were lost to follow up were as follows, respectively: age: 45.25 (±6.67) vs 45.07 (±7.15) years, *p* = 0.806; female sex: 57 % vs 59 %, *p* = 0.749; BMI: 27.2 (±4.96) vs 26.8 (±4.31), *p* = 0.499; offspring 52 % vs 47 %, *p* = 0.891; knee ROA: 18 % vs 15 %, *p* = 0.486 and total tibiofemoral cartilage volume at baseline (mm^3^): 14199 (±3463) vs 14113 (±3410), *p* = 0.611.

Table 1 describes the baseline characteristics of the group stratified by the mean total cartilage volume loss over 10 years. The average age of the cohort was 45 years. Participants with greater than the mean absolute volume loss were significantly older, had a significantly lower percentage of male participants, a significantly higher prevalence of medial JSN, medial osteophytes and any meniscal tear, and a higher medial and lateral tibiofemoral cartilage volume at baseline visit.

**Table 1:** Baseline characteristics of participants split by total (tibiofemoral) cartilage loss (absolute) over 10 years ^a^

	Total volume loss	Total volume loss	*P*-Value
	<16 % (*n* = 109)	≥16 % (*n* = 110)	
Age (years)	**44.2(6.9)**	**46.2(6.6)**	**0.038**
Males (%)	**69**	**45**	**0.001**
BMI	26.8(4.4)	27.3(5.2)	0.449
Any medial JSN (%)	**8**	**20**	**0.015**
Any lateral JSN (%)	2	3	0.659
Any medial osteophytes (%)	**3**	**12**	**0.016**
Any lateral osteophytes (%)	5	6	0.770
Medial (tibiofemoral) cartilage volume (mm^3^)	**6098 (1431)**	**7435 (1583)**	**<0.001**
Lateral (tibiofemoral) cartilage volume (mm^3^)	**6577 (1716)**	**7969 (1757)**	**<0.001**
Total (tibiofemoral) cartilage defects (mean)	4.0 (1.0)	3.9 (1.3)	0.674
Any meniscal tear (%)	**13**	**31**	**0.005**
Any meniscal extrusion (%)	5	15	0.079
Any (tibiofemoral) BMLs (%)	54	50	0.597

^a^Mean (SD) except for percentages. *P*-values determined by *t*-test or *x* ^2^ test (where appropriate)

Bold font signifies statistically significant results

Both absolute and percentage per annum cartilage volume loss were higher in the medial tibiofemoral compartment compared to the lateral tibiofemoral compartment. Mean absolute and percentage per annum cartilage volume loss was 1284 mm^3^ and 1.91 % respectively in the medial compartment and 1007 mm^3^ and 1.38 % respectively in the lateral compartment.

**Fig. 1:**
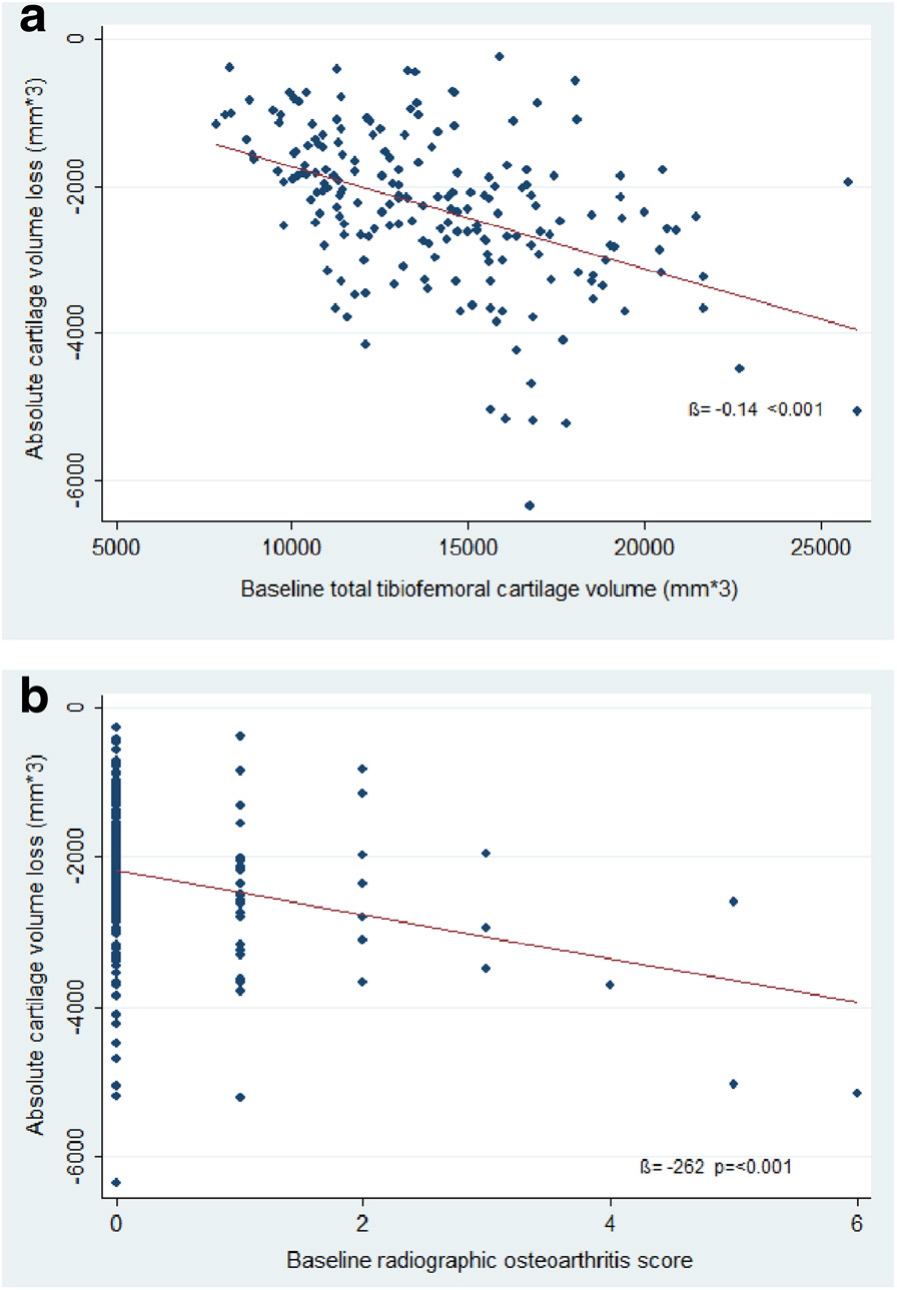
Association between baseline tibiofemoral cartilage volume/ROA score and absolute cartilage volume loss over 10 years

Figure 1a describes the association between baseline tibiofemoral cartilage volume and absolute cartilage volume loss. A higher baseline cartilage volume was associated with higher absolute cartilage volume loss over 10 years. Figure 1b describes the association between baseline ROA score and absolute cartilage volume loss. ROA score ranged from 0 to 6 (possible range 0–18) in the study population at the baseline visit. A higher baseline ROA score was associated with higher absolute cartilage volume loss on average over 10 years.

Table 2 describes the association between the baseline tibiofemoral cartilage volume and cartilage volume loss over 10 years. Baseline cartilage volume was significantly associated with absolute cartilage volume loss over 10 years in both compartments in the multivariable analysis. Further adjustment for cartilage defects and BMLs did not change the effect size considerably. There was a similar trend for the association between the baseline tibiofemoral cartilage volume and percentage per annum cartilage volume loss but the association reached statistical significance in the lateral compartment only (β (95 % CI) = −0.15 (−0.29, −0.02), *p* = 0.01) in the fully adjusted model.

**Table 2:** Association between baseline cartilage volume and cartilage volume loss over 10 years

	Unadjusted	Adjusted^a^
Baseline cartilage volume (site)	β (95 % CI)	β (95 % CI)
Medial (tibiofemoral) cartilage volume loss (absolute)		
Medial tibiofemoral (per SD)	**−265 (−337,−194)**	**−300 (−399,−200)**
Lateral (tibiofemoral) cartilage volume loss (absolute)		
Lateral tibiofemoral (per SD)	**−226 (−303,−148)**	**−338 (−443,−233)**

^a^Adjusted for age, sex, BMI, offspring-control status, ROA at baseline, meniscal tears at visit-2 and meniscal extrusion at baseline where appropriate

*SD* Standard deviation

Bold denotes significant results

Sub-group analysis was done to describe the association between baseline cartilage volume stratified by mean volume and absolute cartilage volume loss over 10 years. Both greater than or equal to the mean total tibiofemoral cartilage volume (β (95 % CI) = −744 (−1162, −325)) and less than mean cartilage volume (β (95 % CI) = −423 (−837, −9)) significantly predicted cartilage volume loss in the fully adjusted model.

Table 3 describes the association between the baseline radiographic measures and cartilage volume loss over 10 years. There were significant associations between medial JSN, lateral JSN and osteophyte scores at baseline and compartment specific absolute cartilage volume loss in the unadjusted analysis. However, none of these associations persisted in the multivariable analysis. Similarly there were no significant associations between the baseline radiographic measures and percentage per annum cartilage volume loss in either the unadjusted or the fully adjusted models.

**Table 3:** Association between baseline radiographic measures and cartilage volume change over 10 years

	Unadjusted	Adjusted^a^
Baseline radiographic measure	β (95 % CI)	β (95 % CI)
Medial (tibiofemoral) cartilage volume loss (absolute)		
Medial JSN (per SD)	**−124 (−204,−43)**	−77 (−170,+14)
Medial osteophytes (per SD)	−71 (−150,+8.9)	+61 (−20,+143)
Lateral (tibiofemoral) cartilage volume loss (absolute)		
Lateral JSN (per SD)	**−80 (−158,−2)**	−21 (−109,+67)
Lateral osteophytes (per SD)	**−172 (−246,−97)**	−72 (−175,+32)

^a^Adjusted for age, sex, BMI, offspring-control status, cartilage volume at baseline, meniscal tears at visit-2 and meniscal extrusion at baseline and/or JSN and osteophytes at baseline where appropriate

*SD* Standard deviation

Bold denotes significant results

Further sub-analyses looking at the association between baseline cartilage volume and/or ROA and absolute cartilage volume loss stratified by mean age showed no significant differences between the two age groups except for a significantly stronger association between baseline cartilage volume and cartilage volume loss over 10 years in the lateral compartment only in the older participants. Participants with mean age ≥45 years showed a significant association (β = −405 (−558, −252)) between the baseline lateral tibiofemoral cartilage volume and cartilage volume loss, whereas participants with mean age <45 years showed no significant association (β = −173 (−387, +41)).

Analyses to explore interactions between the offspring and control groups found no statistically significant difference between the two groups for any of the associations described above. The association between baseline cartilage volume and cartilage volume loss were statistically significant in both the offspring and controls groups when analysed separately (data not shown).

## Discussion

This longitudinal study documents the associations between baseline cartilage volume/ROA and cartilage volume loss over 10 years. Mean absolute and percentage per annum cartilage volume loss was substantial (19.1 % and 13.8 % in the medial and lateral compartments over 10 years) but less than that seen in older populations [8]. Higher baseline tibiofemoral cartilage volume independently predicted greater absolute cartilage volume loss in both compartments and percentage per annum loss in the lateral compartment only. Baseline JSN and osteophytes did not independently predict absolute or percentage per annum cartilage volume loss in either compartment.

This is the first study to describe an independent association between baseline cartilage volume and absolute cartilage volume loss over 10 years. Some recent studies have shown similar associations between baseline cartilage volume and cartilage volume loss over shorter timeframes but none of these studies accounted for knee structural abnormalities such as meniscal tears, meniscal extrusion and BMLs [2, 26, 27]. All of these structures have been shown to predict cartilage volume loss [28] and are potential confounders for the associations described in this study. The association was independent of these factors in the current study. Furthermore, none of the studies mentioned above described the association between the baseline cartilage volume and cartilage volume loss for both tibial and femoral sites.

A criticism of identifying people who will lose more cartilage using the baseline cartilage volume is that association could be due to regression to the mean/tracking. Regression to the mean is a statistical phenomenon that can make natural variation in repeated data look like real change [29]. It happens when unusually large or small measurements tend to be followed by measurements that are closer to the mean. Unusually high or low cartilage volume to begin with could be due to a number of factors such as cartilage random variation due to body size, sex and co-pathologies. To counter the effect of regression to the mean, further sub-group analysis was done with baseline cartilage volume stratified by the mean value. Both group showed a significant association between the baseline cartilage volume and cartilage volume loss, albeit with a greater effect size in participants with a higher baseline cartilage volume. This suggests that the significant association we described is not solely due to regression to the mean. Similar independent association in the lateral compartment for percentage per annum loss, which also takes into account the cartilage volume to begin with, also suggests that this association is real. However, we did not see any independent associations for medial compartment percentage per annum loss suggesting there is an increase in cartilage volume, due to cartilage swelling, that precedes cartilage volume loss in early OA [7]. Early OA is characterised by matrix changes including a reduction in cellular and proteoglycan content and subsequent water retention and proteoglycan dilution [30]. This depletion of proteoglycan matrix has been closely related to the progression of OA [9]. The swelling of cartilage, in the form of increased volume [9], detected by MRI in early OA has been shown to correlate with depletion proteoglycan matrix and cartilage volume loss, and would explain the associations described in this study.

Few longitudinal studies have looked at the association between baseline ROA and cartilage volume loss and to date, they have shown mixed results. Preliminary cross-sectional findings published from this cohort showed that JSN but not osteophytes were associated with a decreased tibial cartilage volume [18]. Similarly Saunders et al. [10] examined the relationship in a randomly selected older cohort over three years and found that JSN and osteophytes both predicted volume loss in a dose response manner but did not adjust for potential confounders such as meniscal tears/extrusion and BMLs. Furthermore, studies looking at the association between JSN and cartilage thickness loss have shown mixed results as well [20, 31], possibly due to different study populations and shorter follow-up periods. Univariable analysis looking at the association between baseline ROA and cartilage volume loss from our 10 year data showed similar results to Saunders et al. [10] but none of these associations persisted once adjusted for MRI assessed co-pathologies. JSN is a composite of structures that are not visible on radiographs. Variation in JSN and longitudinal changes are a reflection of changes in cartilage and meniscus [32]. Hence when adjusted for abnormalities in these structures, JSN did not independently predict cartilage volume loss. Osteophytes are considered an instigating factor in OA causal pathway and studies have shown that presence of osteophytes is associated with a higher prevalence of cartilage defects and decreased cartilage volume. However, once adjusted for co-pathologies, osteophytes failed to independently predict cartilage volume loss in either compartment. Recent studies have suggested that loss of meniscal function is associated with both cartilage volume loss and presence of osteophytes due to increased bio-mechanical stress on the underlying cartilage and the bone. These results and the data from the present study suggest that osteophytes may be on the OA causal pathway or an attempt at repair and are probably not an independent instigating factor for early cartilage volume loss.

Rate of cartilage volume loss and OA progression varies from patient to patient. Cartilage volume loss is often the end-point in chondro-protective drug trials and has been shown to predict total knee replacement surgery [22]. It is imperative for chondro-protective trials to identify fast progressors to make these trials more responsive and sensitive to change. Previous studies have suggested that degree of JSN can be used to identify the sub-groups, which will lose cartilage faster in chondro-protective drug trials [31]. However, data from this study shows that JSN is not an independent predictor of cartilage volume loss. On the other hand, our results suggest that cartilage volume to begin with can be used to identify fast progressors especially if we can differentiate between swollen and non-swollen cartilage.

The key strength of this study is the long follow up period. To our knowledge this study has the longest follow up period using MRI to monitor disease progression in OA. Another strength is that we examined both femoral and tibial cartilage volume loss whereas previous studies have often only reported on one or the other. Lastly, adjustment for other MRI structural co-pathologies points towards the mediating mechanisms involved in cartilage volume loss. This study has a number of limitations as well. First, around 40 % of participants were lost to follow up at ten years. Those lost to follow-up however were found to be similar in terms of baseline characteristics compared to the participants who were followed-up. Secondly we examined a specific middle-aged group and therefore the results cannot be generalised to the entire population especially people with advanced OA. We believe our results are generalisable to a middle-aged population as we did not see any significant differences between the offspring and control groups for any of the associations described in this study. Third, meniscal tears and BMLs were scored at two years and not at the baseline visit. However changes in these structures over 8 years was small suggesting that these are unlikely to change the effect size considerably.

## Conclusion

Cross-sectional cartilage volume measurement independently predicts cartilage volume loss over 10 years and can be used to identify fast progressors in clinical trials. Radiographic JSN and osteophytes on the other hand are a reflection of other co-pathologies assessed on MRI and do not independently predict cartilage volume loss over 10 years.
